# Sociodemographic and early-life predictors of being overweight or obese in a middle-aged UK population– A retrospective cohort study of the 1958 National Child Development Survey participants

**DOI:** 10.1371/journal.pone.0320450

**Published:** 2025-03-26

**Authors:** Glenna Nightingale, Karthik Mohan, John Frank, Sarah Wild, Sohan Seth

**Affiliations:** 1 School of Health in Social Sciences, University of Edinburgh, Edinburgh, United Kingdom; 2 School of Informatics, University of Edinburgh, Edinburgh, United Kingdom; 3 Usher Institute, University of Edinburgh, Edinburgh, United Kingdom; Christian Medical College, INDIA

## Abstract

Obesity has become a global public health concern. However, its precise origins and causation are still hotly debated, especially the relative importance of individual-level genetics and behaviours, as opposed to obesogenic environmental factors. Our key objective is to quantify the impact of sociodemographic and early-life course predictors of being overweight or obese at 16, being overweight/obese/severely obese42 years of age, and on the incidence of a status of being overweight/obese/severely obese between 16 and 42 years of age, spanning the years before and after marked increases in obesity prevalence in the UK. We used data collected from participants and their mothers from the 1958 National Child Development Survey. The outcomes of interest were being overweight (defined as $25kg/m^{2}<BMI\leq 29.9kg/m^{2}$ ) or obese (defined as BMI > 30 kg/m2) at 16 and 42 years of age and incident obesity between 16 and 42 years of age. We assessed the risk factors for obesity using logistic regression models. We observed a strong influence of maternal obesity for being Obese/Severe Obese compared to being overweight across the three models (ORs 4.328,2.901,3.293 for the models relating to age 16, the age range 16-42, and age 42 respectively). Additionally, we note that maternal smoking (ORs 1.6 to 1.8 for 10 + cigarettes per day compared to non-smokers) on all three outcomes were statistically significant. Females were prone to being overweight/obese at 16 years of age (OR 1.96 CI 1.61 to 2.39) but less prone to develop obesity between 16 and 42 years of age (OR 0.89 CI 0.78 to 1.007). Our results suggest that sociodemographic and early-life risk factors could be used to target obesity prevention programmes for children and adults. In particular, we note that the effect of maternal influences persists through to age 42 and that strikingly, those predictors were just as powerful (and prevalent) in the era before the current obesity pandemic began. This suggests that, as Geoffrey Rose pointed out, novel studies are needed of factors at the community/societal level that may have caused the current obesity pandemic, since individual-level risk factors appear not to have changed over the time period spanning the pandemic’s onset and growth.

## Introduction

The public health concern of obesity and, to a lesser degree, overweight, is a critical research priority globally [1–4] Obesity increases the risk of cardiovascular disease, osteoarthritis, type 2 diabetes, and some cancers [5]. Addressing the obesogenic environment and promotion of healthy behaviours in individuals form components of public health intervention. In recent years, the life course approach [6] has demonstrated that maternal factors, early-years characteristics and socio-economic status at all stages of life are associated with weight-for-height, i.e., body mass index (BMI) trajectories. This study uses the National Child Development Survey (NCDS), also known as the 1958 British Cohort Study, (Power & Elliott, 2006) to investigate the life-course determinants of obesity among individuals during whose lifetime the obesity “pandemic” has developed in the UK. The NCDS begun in 1958 and has been following individuals born in a single week of March 1958 to present. There have been 10 sweeps so far and the last sweep was in 2020 where the cohort members were 62 years of age. For the first survey sweep, the total number of individuals surveyed was 17,415 and were born in either England, Scotland or Wales – this has been reported to be 98% of all births across England, Scotland and Wales.

The childhood experiences of the cohort were different from that experienced by children in the 2020s [7] and the levels of obesity were not as high as at present. During the childhood period of the cohort, most households lacked basic facilities, and breast-feeding and maternal smoking were common.

Notably, the cultural and physical environments in the UK, especially features related to access to and consumption of food and facilitators of/barriers to physical exercise habits, have changed markedly over the cohort’s lifetime.

We were able to identify only one study [8] that uses the NCDS dataset to assess the influence of childhood life-course risk factors on obesity through multivariate analysis, using data from 11,752 participants with BMI measurements at 23, 33 and 42 years of age as outcome. The study only investigated the influence of four variables, i.e., low or high birthweight; never breastfed; physical activity at age 11; and at least one obese parent at age 11. The only other relevant primary study is based on a much smaller (n = 762) Dutch cohort, modelling BMI in a cohort born between 1977 and 1986 (when the obesity pandemic had already begun) [9]. This study only modelled BMI at three ages between 2 and 6 years as continuous variables. Other recent studies [10] using multiple risk factors in the Growing Up in Scotland Cohort data [11,12]and using Born in Bradford Cohort data [13] have used more potential risk factors and performed better in predicting later-onset of obesity as measured by AUC-ROC.

Our study models the influences of not only early-life predictors but also socioeconomic factors such as father’s social class. Our study also uses exercise frequency at age 33, which is the only “lifestyle” risk factor on which adequately complete data were available (with only 13.28% data missing).

The epidemiological association, between maternal characteristics and childhood obesity is well established [2,14,15]. Smoking during pregnancy, maternal BMI and parental socioeconomic status have frequently been implicated as risk factors for obesity in the offspring. For example, mothers’ smoking during pregnancy was weakly associated (OR 1.56 CI 1.13 to 2.15) with obese growth trajectories in children [2,15].

Low birthweight (LBW) has been linked (Oken , 2009) to obesity, and adult chronic health conditions [16,17]. However, absence of reliable gestational age data at the time of our cohort’s birth, i.e., well before ultrasound was available, precluded analysing small-for-gestational age subjects separately from premature births, which we felt precluded sensible interpretation of such an analysis. However, we did examine whether the three obesity outcomes were associated with lower birth weight, but have not presented the results in this paper.

### Aims

Our aim was to describe the sociodemographic and early-life predictors of being overweight or obese (O16) at 16 years of age, of being overweight, obese or severely obese at 42 years of age(O42), and additionally of accruing obesity between 16 and 42 years of age. That is, not being overweight nor obese at 16 years of age but being overweight or obese or severely obese at 42 years of age (O16-42). Note that in our study, the BMI categories underweight, healthy, overweight, obese and severely obese are categorized by BMI<18.5, 18.5≤BMI≤25, 25<BMI≤29.9, 30>BMI≤40, and BMI>40respectively, where BMI is measured in kg/m^2^. The corresponding research questions are:

- What are the sociodemographic and early-life predictors of being overweight/obese at 16 years of age **[O16]**?
- What are the sociodemographic and early-life predictors of being overweight/obese/severely obese at 42 years of age **[O42]**?
- What are the sociodemographic and early-life predictors of not being overweight/obese at 16 years of age but being obese or severely obese at 42 years of age **[O16-42]?**

#### Data variables used in this study.

We conducted a retrospective cohort study using the NCDS [7] dataset on a cohort of individuals all born within the same week in 1958. Data have been collected 12 times between 1958 and 2020 with each data collection known as a ‘sweep’. Notably, because this cohort’s members were all born in the same week of 1958, we do not need to account for period (calendar-time) effects. The dataset contained information at 0, 7, 11, 16, 23, 33, 42, 44, 46, 50, 55, and 62 years of age. For this project, we used data collected at 0, 7, 11, 16, 33 and 42 years of age (corresponding to sweeps 0, 1, 2, 3, 4, 5 and 6 respectively) [18].

The age of 42 years was chosen because of limited missingness and to provide data for early middle age – prior to the perimenopause for most women. Additionally, the age of 16 was chosen to compare early and middle adulthood. Of the data sweeps available in our dataset, the one at age 16 was closest to what most people think of as “the end of childhood”. At age 16, female members, would have already experienced menarche and the potential weight fluctuations associated with puberty.

The data variables considered in this study are BMI (participant’s mother at birth, and participant at 16 and 42 years of age), exercise frequency at 33 years of age, method of delivery at birth, father’s social class at birth, participant’s birth weight, birth order, whether or not the mother smoked at birth, age of mother at birth, and participant’ sex. The reference/comparison group for each of the categorical variables was guided by reported associations for these variables observed in the literature such as in (Doi et al., 2016).

The exercise frequency at 33 years of age provides an indication of the impact of exercise on obesity incidence (in subsequent years, leading up to age 42, when BMI was measured). Variables such as father’s social class, participant’s sex, birth weight and birth order were considered to incorporate socio-demographically important factors into the analysis.

Further details on the data sweeps and variables used in this study can be found in S1 Table1 and S2 Table 2 in S1 File, respectively. The data transformations employed in this study are detailed in S3Text, S4 Table 3, S5 Text, S6 Text, S7 Table 4, S8 Text, S9 Text, and S10 Table 5 in S1 File).

## Methods

### Data missingness and anomalies

After excluding records for which the predictors of interest were missing, 6,933 individuals had data available for O42. Among these individuals, 5,831 individuals had both height and weight available at 16 years of age and were used for O16. Among these individuals, 4,767 individuals were not overweight at 16 years of age and were used for O16-42.

Finally, in sweep 6 we identified outliers due to missing values being encoded as a high value (9999 or similar). We also excluded values in the birthweight variable which were only estimates due to low accuracy.

**Fig 1:**
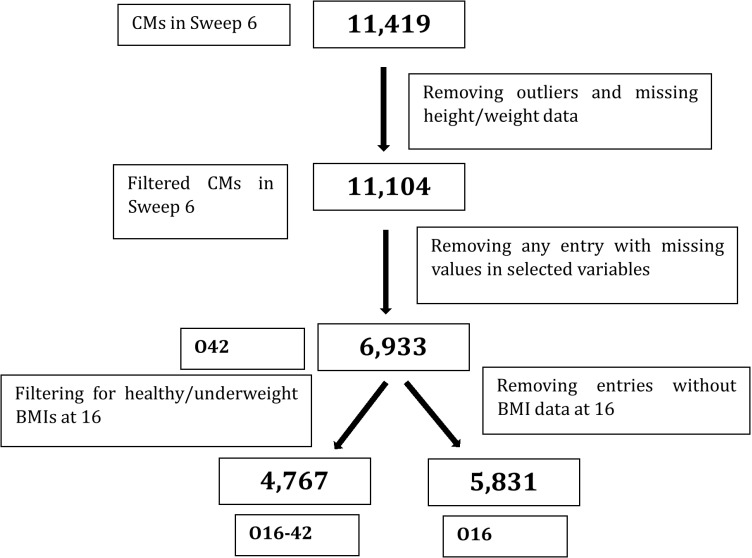
Flow diagram depicting the sample size for each research question.

Fig 1 depicts the process followed. The characteristics of key variables (xsee S11 Figure, S12 Figure, and S13 Figure in S1 File) for the complete cases dataset of 6,933 were similar to those of the full dataset of 11,419.

#### Exploratory analyses and inferential statistics.

We summarised the variables used in our study in Table 1. Our modeling approach involves using logistic regression to describe the associations between risk factors of being overweight/obese at 16 years of age (O16), of being overweight, obese/severely obese at 42 years of age (O42) and being overweight/obese/severely obese at 42 years of age for individuals who are healthy or underweight at 16 years of age (O16-42). The predictors of the models considered are outlined in Table 1.

**Table 1:** Predictors and outcome for each model.

		Overweight/Obese at age 16 years	Overweight/Obese/Severely Obese between ages 16 and 42 years	Overweight/Severely Obese/Obese at 42 years of age
Sociodemographic predictors	Father’s social class	x		
	Job category at age 42		x	x
Early-life predictors	Mother’s smoking at birth	x	x	x
	Delivery method	x	x	x
	Sex	x	x	x
	BMI of mother at birth	x	x	x
	Birth order	x	x	x
	Age of Mother at birth	x	x	x
Lifestyle/chronic disease risk factor	Exercise frequency at age 33		x	x

The predictors used for the model O42 are based on the results of stepwise elimination of predictors, starting with those with the least significant p-values under p = 0.05, informed by two considerations: a) AIC change: if the AIC was reduced by at least 2, the predictor was retained in the model under consideration, and b) any predictor which caused a change of more than 10% in any other predictors’ coefficient was retained [19], as there is evidence that it is a confounder of important effects (on the outcome variable). The selected predictors were also used for O16-42. For O16, the socio-economic status was indicated using father’s social class instead of using the participant’s job category. Additionally, in O16, exercise at age 33 was not used since this variable was not available at age 16.

Ethics approval for our study was obtained from the Ethics committee of the Nursing Studies department of the School of Health in Social Studies of the University of Edinburgh. The Ethics application ID is 22-23NUST021. This study did not involve collection of data in any form. All the data used in this study was obtained from the publicly available NCDS longitudinal dataset. This data is publicly available from the UK Data Service and the use of this data does not require obtaining consent from the participants of the NCDS study. The NCDS is 62 years old and details on the consent protocol followed by the NCDS can be found here: https://cls.ucl.ac.uk/wp-content/uploads/2017/07/NCDS-Ethical-review-and-Consent-2014.pdf.

## Results

### Exploratory analyses

**Fig 2:**
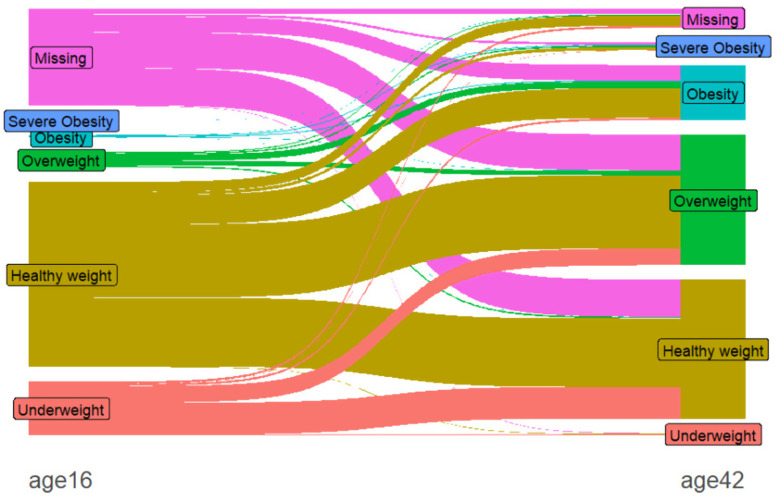
Sankey diagram depicting the BMI categories across the two sweeps corresponding to 16 and 42 years of age.

Fig 2 (Sankey diagram) shows a striking difference in the BMI category profiles at 16 and 42 years of age. Interestingly, the proportion of individuals who were overweight at 42(36.96%) is similar to that of those who were of healthy weight (39.35%) at the same age. In contrast, at age 16, the majority of individuals were of healthy weight (52.43%), and a relatively small proportion of individuals were overweight (4.23%).

Table 2 provides summaries of the variables used in our study with percentages per level (and sample size). From Table 2 we note that for model [O16-42] in terms of Exercise frequency at age 33, the higher percentages of individuals who did not develop obesity were for those who exercised 4-5 times a week or every day.

**Table 2:** Descriptives for the variables used in the study.

Variables	Levels	Overweight/Obese at age 16 years		Overweight/Obese/Severely Obese between ages 16 and 42 years		Overweight/Severely Obese/Obese at 42 years of age		Full Cohort	
		N	%	N	%	N	%	N	%
**Father’s social class (ref. Others)**	Farm	238	40.614						
	Manual	147	25.085						
	Others	28	4.778						
	Professional	173	29.522						
**Job category at age 42 (ref. Others)**	Others			173	17.129	384	18.003		
	Professional/Technical/Non-Manual			496	49.109	1028	48.195		
	Skilled/Unskilled Manual			341	29.27	721	33.802		
**Mother’s smoking at birth (ref. Does not smoke)**	Does not smoke	229	39.079	420	41.584	645	32.774	3876	38.034
	Less than 1 a day	14	2.389	20	1.98	31	1.5752	255	2.502
	1-10 per day	95	16.212	153	15.149	260	13.211	1241	12.177
	10+ per day	184	31.399	275	27.228	467	23.729	1964	19.272
	Unknown/Others	64	10.922	142	14.059	565	28.709	2855	28.015
**Delivery method (ref. Vertex and hand)**	Vertex and hand	488	83.276	857	84.851	1670	84.858	8794	86.292
	Caesarean-labour	14	2.389	21	2.079	36	1.8293	145	1.423
	Caesarean-elect	9	1.536	13	1.287	23	1.169	117	1.148
	Others	75	12.799	119	11.782	239	12.144	1135	11.137
**Sex (ref. Male)**	Male	208	35.495	567	56.139	1017	51.677	5039	49.446
	Female	378	64.505	443	43.861	951	48.323	5152	50.554
**BMI of mother at birth (ref. Healthy)**	Healthy	236	46.094	484	55.125	847	52.059	5596	65.611
	Obese/Severe Obese	94	18.359	106	12.073	230	14.136	631	7.398
	Overweight	169	33.008	264	30.068	506	31.1	1945	22.805
	Underweight	13	2.539	24	2.733	44	2.704	357	4.186
**Exercise Frequency at age 33**	No exercise			226	25.028	505	27.165		
	Less often			27	2.99	46	2.474		
	2-3 times a month			65	7.198	126	6.778		
	Once a week			199	22.038	395	21.248		
	2-3 days a week			183	22.989	361	19.419		
	4-5 days a week			51	5.648	94	5.056		
	Every day/most days			152	16.833	332	17.859		

### Models

**Fig 3:**
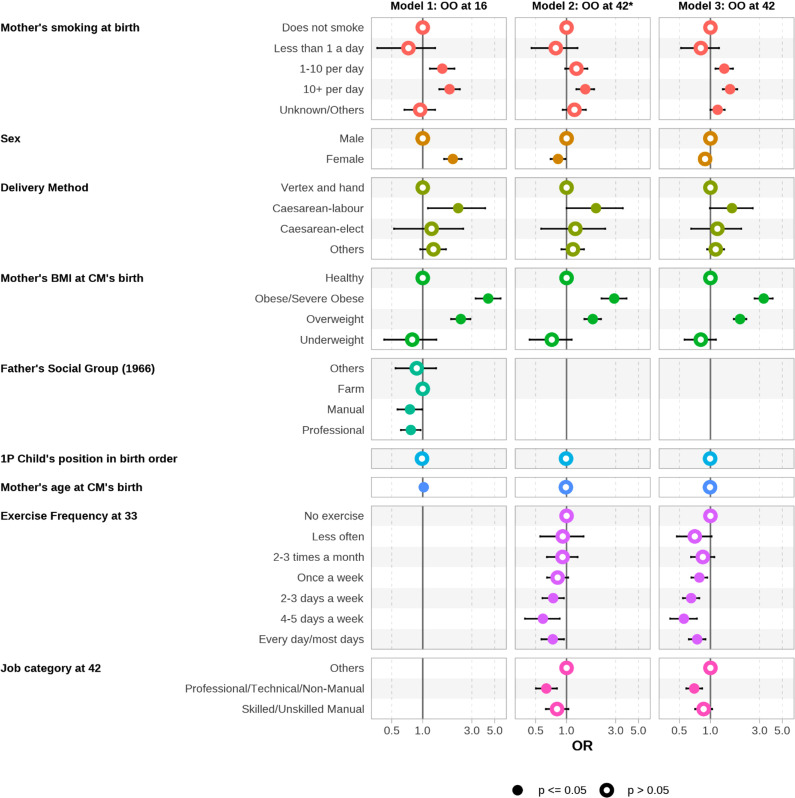
Forest plots for (left) odds of being overweight/obese at age 16 denoted as [O16], (middle) odds of being overweight/obese/severely obese at age 42 for those who were healthy/underweight at age 16 denoted as [O16-42], and (right) odds of being obese/overweight at age 42, denoted as [O42].

Here we present in Fig 3, the results for the three models **[O16], [O16-42]**, and **[O42]** considered in this study. Table 3–5 provide details on the odds ratios (OR) for each model. Note that the outcome variable for [**O16]** is being overweight or obese at 16 years of age, that for **[O16-42]** is being healthy at 16 years of age but overweight, obese or severely obese at 42 years of age, that for [**O42]** is being overweight, obese or severely obese at age 42.

**Table 3:** Results for Obesity at 16 years of age (n = 5831, 33.261% of N).

Characteristic	OR1	95% CI	p-value
Mother’s smoking at birth			
Does not smoke	—	—	
Less than 1 a day	0.726	0.361, 1.322	0.33
1-10 per day	1.549	1.171, 2.034	**0.002**
10+ per day	1.822	1.446, 2.294	**<0.001**
Unknown/Others	0.945	0.662, 1.320	0.746
Sex			
Male	—	—	
Female	1.961	1.612, 2.392	**<0.001**
Delivery Method			
Vertex and hand	—	—	
Caesarean-labour	2.21	1.122, 4.052	**0.015**
Caesarean-elect	1.219	0.526, 2.474	0.611
Others	1.271	0.945, 1.684	0.103
Mother’s BMI at CM’s birth			
Healthy	—	—	
Obese/Severe Obese	4.328	3.261, 5.711	**<0.001**
Overweight	2.344	1.886, 2.907	**<0.001**
Underweight	0.791	0.422, 1.359	0.428
Father’s Social Group (1966)			
Farm	—	—	
Manual	0.752	0.568, 0.985	**0.042**
Others	0.875	0.544, 1.349	0.561
Professional	0.766	0.614, 0.954	**0.017**
Child’s position in birth order	0.983	0.916, 1.053	0.631
Mother’s age at CM’s birth	1.023	1.003, 1.043	**0.024**
Log-likelihood	-1,562		
AIC	3,158		
BIC	3,271		
Nagelkerke R²	0.09		

OR =  Odds Ratio, CI =  Confidence Interval.

**Table 4:** Results for Obesity at 42 years old for individuals healthy/underweight at 16 years old (n = 4767, 27.192% of N).

Characteristic	OR1	95% CI	p-value
Exercise Frequency at 33			
No exercise	—	—	
Less often	0.917	0.554, 1.463	0.727
2-3 times a month	0.914	0.643, 1.280	0.607
Once a week	0.818	0.645, 1.037	0.097
2-3 days a week	0.741	0.582, 0.942	**0.014**
4-5 days a week	0.589	0.394, 0.859	**0.008**
Every day/most days	0.734	0.569, 0.944	**0.017**
Job category at 42			
Others	—	—	
Professional/Technical/Non-Manual	0.635	0.503, 0.806	**<0.001**
Skilled/Unskilled Manual	0.808	0.628, 1.043	0.099
Mother’s smoking at birth			
Does not smoke	—	—	
Less than 1 a day	0.785	0.455, 1.278	0.355
1-10 per day	1.247	0.968, 1.595	0.083
10+ per day	1.519	1.243, 1.854	**<0.001**
Unknown/Others	1.193	0.916, 1.542	0.183
Delivery Method			
Vertex and hand	—	—	
Caesarean-labour	1.931	0.998, 3.521	**0.039**
Caesarean-elect	1.216	0.566, 2.374	0.589
Others	1.153	0.891, 1.478	0.269
Sex			
Male	—	—	
Female	0.826	0.699, 0.975	**0.024**
Mother’s BMI at CM’s birth			
Healthy	—	—	
Obese/Severe Obese	2.901	2.184, 3.829	**<0.001**
Overweight	1.797	1.490, 2.162	**<0.001**
Underweight	0.718	0.437, 1.123	0.167
Child’s position in birth order	0.988	0.925, 1.053	0.713
Mother’s age at CM’s birth	0.984	0.967, 1.001	0.073
Log-likelihood	-1,960		
AIC	3,964		
BIC	4,106		
Nagelkerke R²	0.054		

OR =  Odds Ratio, CI =  Confidence Interval.

**Table 5:** Results for obesity at 42 years old (n =  6933, 39.547% of N).

Characteristic	OR1	95% CI	p-value
Exercise Frequency at 33			
No exercise	—	—	
Less often	0.705	0.471, 1.029	0.079
2-3 times a month	0.844	0.647, 1.093	0.203
Once a week	0.781	0.653, 0.933	**0.006**
2-3 days a week	0.65	0.540, 0.780	**<0.001**
4-5 days a week	0.552	0.407, 0.739	**<0.001**
Every day/most days	0.744	0.616, 0.897	**0.002**
Job category at 42			
Others	—	—	
Professional/Technical/Non-Manual	0.696	0.583, 0.833	**<0.001**
Skilled/Unskilled Manual	0.859	0.710, 1.041	0.119
Mother’s smoking at birth			
Does not smoke	—	—	
Less than 1 a day	0.806	0.518, 1.213	0.319
1-10 per day	1.364	1.119, 1.658	**0.002**
10+ per day	1.552	1.319, 1.825	**<0.001**
Unknown/Others	1.175	1.000, 1.380	0.049
Delivery Method			
Vertex and hand	—	—	
Caesarean-labour	1.62	0.987, 2.577	0.048
Caesarean-elect	1.169	0.650, 1.997	0.584
Others	1.126	0.928, 1.359	0.223
Sex			
Male	—	—	
Female	0.887	0.782, 1.007	0.063
Mother’s BMI at CM’s birth			
Healthy	—	—	
Obese/Severe Obese	3.293	2.685, 4.032	**<0.001**
Overweight	1.942	1.686, 2.234	**<0.001**
Underweight	0.806	0.559, 1.133	0.231
Child’s position in birth order	0.993	0.947, 1.040	0.768
Mother’s age at CM’s birth	0.991	0.978, 1.004	0.162
Log-likelihood	-3,258		
AIC	6,560		
BIC	6,711		
Nagelkerke R²	0.066		

OR =  Odds Ratio, CI =  Confidence Interval.

The associated sample sizes for these models are: 5,831, 4,767, and 6,933 respectively, as described in Fig 1

We have provided results for the full model in S15 Table in S1 File.

## Discussion

We begin the discussion by addressing the study’s research questions.

**Research question 1 [O16]**: We found associations between sex, maternal factors (BMI, smoking, mode of delivery), father’s socio-economic position and being overweight at 16 years of age (in 1974). Higher odds ratios (see Table 3) were observed for females, for increasing maternal age, for mothers who smoked 10 cigarettes or more per day (compared to those who did not smoke), for caesarean-labour-delivery (compared to normal delivery), and for maternal obesity (compared to the mothers being healthy or underweight). The odds ratio (4.33, CI 3.26 to 5.71) for maternal obesity is the largest amongst the variables considered. Father’s socioeconomic position showed modest protective effects for “Manual” and “Professional” categories compared to “Farming” category, and birth order was not associated with obesity at 16 years of age.

With regards to the finding on caesarean-labour-delivery, one possible explanation is that mothers who are overweight and with gestational diabetes often require C-sections and their babies are likely to be overweight. This predisposes the babies to later weight gain [20–22].

**Research question 2 [O16-42]**: Similar risk factors were associated with incident obesity between 16 and 42 years of age, albeit with generally slightly smaller odds ratio, compared to those described above for obesity at 16 years of age, although the association with sex changed direction so that being male was a risk factor for incident obesity between 16 and 42 years of age. An additional risk factor, exercise frequency at age 33 (with a reference/comparison group of “No exercise”), showed clinical and statistically significant associations (see Table 4) with incident obesity, in a close-to-monotonic dose-response pattern (OR for 2-3 days a week of exercise 0.74, CI 0.58 to 0.94; OR for 4-5 days a week of exercise 0.59, CI 0.39 to 0.85), similarly to findings in previous studies [23–25].

**Research question 3 [O42]**: We note that the estimates for [O16-42] and [O42] for early life and parental risk factors were similar -this may be largely because obesity was so rare at age 16 in 1974, that the adult-onset cases consist mostly of incident obesity between 16 and 42 years of age.

Mother’s BMI and maternal smoking remain important risk factors in O16-42 for adult-onset obesity, between ages 16 and 42 years of age. The persistent importance of maternal factors well into their children’s adult life illustrates the “long reach” of intergenerational influences. The effect of mother’s BMI for example is the largest (OR 3.90, CI 2.69 to 4.03). Details are provided in Table 5.

Geoffrey Rose, considered by some to be the father of modern chronic disease epidemiology, provided a conceptual framework of direct relevance to our findings. In his landmark paper [26] “Sick Individuals and Sick Populations” he provided sage advice to researchers looking for the causes of widespread changes in the prevalence of chronic diseases, such obesity (a topic he addressed directly, even though the modern pandemic was only beginning at that time.) His advice was to suspect a major role for “upstream” risk factors operating at the level of entire societies/communities and to “seek the causes of incidence (at the population level) rather than the causes of cases (at the individual level)”.

Our findings support this strategy in future research into the aetiology of obesity. Specifically, we found remarkably similar relative risks for several early-life risk factors for later-life obesity, spanning a period starting before the obesity pandemic really began (at least in the UK) to well after its peak. Those early-life risk factors predicted, with persistently powerful relative risks, both prevalent obesity at age 16 (rather uncommon in this cohort) and also incident obesity between ages 16 and 42. This finding suggests that such individual-level risk factors are unlikely to explain the pandemic itself. However, to illuminate the role in the pandemic of society/community-level risk factors, those would have to be measured, across societies/communities with varying patterns of emergence for the pandemic over recent decades – ideally in a multi-level study design.

Such designs are not typical of classical epidemiology. Indeed, the British birth cohort we analysed was aimed at the measurement and analysis of only individual- level, not society/community-level risk factors for the many health and development outcomes it collected. If one were to follow Rose’s advice, significant additional data collection in such a cohort study would be needed, reflecting changes in relevant society/community-level risk factors – such as the profoundly transformed nature of food production, marketing and distribution systems between the 1970s through to the early 2000s. We suspect such a novel study design, because it would have to span global national settings with major variation in how and when the pandemic began, would have to be international in scope, perhaps sampling a set of societies with contrasting pandemic patterns of timing – a daunting prospect, but a project we suspect Rose would strongly support.

## Conclusion

The *main strengths* of this study are that our paper is unique in showing that those predictors were just as powerful (and prevalent) in the era before the current obesity pandemic began. This lends great credibility to a central idea of Geoffrey Rose.

Our work supplements previous studies [8,12,27] showing that maternal influences such as BMI, are important predictors of obesity across four decades of offsprings’ lives. Our study also documents persistent sociodemographic influences on obesity over the life course. Additionally, our study is providing a rare glimpse into socio-demographic and early-life influences on obesity onset before and after the appearance of the obesogenic environment in the UK.

One *limitation* is that regrettably, birthweight data collected in 1958 on the NCDS cohort could not include ultrasound estimates of gestational age, making it impossible to separate premature from small-for-gestational-age newborns. After preliminary analyses in which raw birthweight and birthweight adjusted for the mother’s estimated last menstrual period showed no association with the outcomes under study, we decided not to use the birthweight data in our final analyses, despite past studies showing a link between fetal weight and adult obesity.

As indicated in the Introduction, the only study identified [8] which assesses early-life risk factors for obesity in the 1958 British Birth Cohort considers a much narrower range of predictors than our study. Our study confirms the major finding in this paper, pinpointing maternal factors as being important in predicting adult-onset obesity. Our study, however, reveals that other factors such as father’s social class and exercise frequency (at 33 years of age) are also important. The identification of exercise frequency as an important predictor resonates with current public health efforts at promoting wellbeing through physical activity.

Our work has highlighted the importance for future work to investigate the impact of sociodemographic and early-life biological/familial factors’ association with adult obesity, in a cohort whose childhood occurred prior to onset of secular trends in increasing obesity prevalence among both children and adults. The importance for future work of investigating differences in risk factors between obesity in early life, versus adult onset, and especially how this plays out so differently in males and females, has also been shown. Finally, the need for investigating the importance of cultural/environmental conditions in epidemiological studies, not just individual-level characteristics/exposures has been clearly illustrated. Public health officials and researchers (in the 1970s and 1980s) did not anticipate subsequent massive, population-level increases in the prevalence of obesity/overweight, let alone conceptualize the then-emerging obesogenic environment as a key driver of that pandemic. Therefore, it is not surprising that existing cohort datasets from that period do not include credible measures of the “dose,” of specific obesogenic influences within that environment, to which individual subjects were exposed.

The impact of dietary habits on the onset of obesity is one aspect not covered in our analyses. The quality of the available dietary data in the NCDS did not allow for this. Similarly, our analyses did not include the impact of personality traits. In terms of future research, we are planning to conduct a larger study which examines the impact of sociodemographic predictors on the onset of obesity during the 2020s. This study would allow us to determine how different those predictors are in the present era to those observed in the NCDS and inform public health policy regarding health promotion and protection. Another aspect that we will be investigating is the impact of “place”: whether the area where one’s lives in the UK impacts their likelihood of being obese.

This study has identified early-life (and potentially more “biologically mediated”) risk factors for obesity which have been sustained in their importance, for a cohort whose childhood preceded the pandemic, suggesting roles for both biology and environment in obesity time trends.

## Supporting information

S1 Table NCDS data sweeps and corresponding years. (DOCX)

S2 Table Variables used in modelling from NCDS. (DOCX)

S1 Text Data transformations and processing. (DOCX)

S3 Table Transformations for the smoking variable. (DOCX)

S2 Text Method of Actual Delivery. (DOCX)

S3 Text Father, male head’s socio-economic group (GRO 1966). (DOCX)

S4 Table Transformation of father’s occupation. (DOCX)

S4 Text Mother’s BMI at birth. (DOCX)

S5 Text Current Job – Social Class. (DOCX)

S5 Table Transformations for job categories. (DOCX)

S1 Fig Comparison of BMI categories. (DOCX)

S2 Fig Comparison of exercise frequency. (DOCX)

S3 Fig Comparison of mother’s BMI. (DOCX)

S6 Table Model diagnostics. (DOCX)

S7 Table Results for the full model for O42 (with all variables). (DOCX)
