# Supplementary material for: Sociodemographic and early-life predictors of being overweight or obese in a middle-aged UK population– A retrospective cohort study of the 1958 National Child Development Survey participants
Source: PLoS One. 2025 Mar 26;20(3):e0320450. doi: 10.1371/journal.pone.0320450 (PMC11940735; doi:10.1371/journal.pone.0320450)
Supplement: S1 Table — (DOCX) [file pone.0320450.s001.docx]

**Table 1**: NCDS data sweeps and corresponding years

| Sweep | Age of Cohort | Year of data sweep |
| --- | --- | --- |
| 0 | 0 | 1958 |
| 1 | 7 | 1965 |
| 2 | 11 | 1969 |
| 3 | 16 | 1974 |
| 4 | 23 | 1981 |
| 5 | 33 | 1991 |
| 6 | 42 | 2000 |
| 7 | 44 | 2002 |
| 8 | 46 | 2004 |
| 9 | 50 | 2008 |
| 10 | 55 | 2013 |
| 11 | 62 | 2020 |
