# Supplementary material for: Sociodemographic and early-life predictors of being overweight or obese in a middle-aged UK population– A retrospective cohort study of the 1958 National Child Development Survey participants
Source: PLoS One. 2025 Mar 26;20(3):e0320450. doi: 10.1371/journal.pone.0320450 (PMC11940735; doi:10.1371/journal.pone.0320450)
Supplement: S2 Table — (DOCX) [file pone.0320450.s002.docx]

**Table 2**: Variables used in modelling from NCDS.

| Variable | Description | Sweep | Category |
| --- | --- | --- | --- |
| n2400 | No. of cigarettes mother smokes per day | 0 | Early-life |
| n534 | Method of actual delivery | 0 | Early-life |
| n553 | Mother’s age last birthday, in years | 0 | Early-life |
| n1175 | Father, male head’s socio-economic grp (GRO 1966) | 0 | Early-life |
| n1202 | Mother’s weight in stones | 0 | Early-life |
| n1205 | Mother’s height in inches | 0 | Early-life |
| dvwt16 | Weight in kg. at 16 years | 3 | Early-life |
| dvht16 | Height in m. at 16 years | 3 | Early life |
| n504363 | How often undertakes sport/exercise/etc | 5 | Lifestyle |
| htmetre2 | CMs height: metres | 6 | Demographic |
| wtkilos2 | CMs weight: kilos | 6 | Demographic |
| sc | (Current Job) Social Class | 6 | Sociodemographic |
