# Supplementary material for: Sociodemographic and early-life predictors of being overweight or obese in a middle-aged UK population– A retrospective cohort study of the 1958 National Child Development Survey participants
Source: PLoS One. 2025 Mar 26;20(3):e0320450. doi: 10.1371/journal.pone.0320450 (PMC11940735; doi:10.1371/journal.pone.0320450)
Supplement: S1 Text — (DOCX) [file pone.0320450.s003.docx]

**Data transformations and processing**

BMI at 16: BMI category at 16 years age was defined using WHO guidelines. For males, the categories are underweight (<18.7 kg/m2), healthy (18.7 - 24.2 kg/m2), overweight (24.2 - 28.6 kg/m2), and obese (> 28.6 kg/m2). For females, the categories are underweight (< 18.4 kg/m2), healthy (18.4-24.5 kg/m2), overweight (Between 24.5 and 29.3 kg/m2), and obese (> 29.3 kg/m2).

Smoking: The number of cigarettes smoked by a given cohort member’s mother was recoded as shown in Table 3 to overcome excessively small cells.
