# Supplementary material for: Sociodemographic and early-life predictors of being overweight or obese in a middle-aged UK population– A retrospective cohort study of the 1958 National Child Development Survey participants
Source: PLoS One. 2025 Mar 26;20(3):e0320450. doi: 10.1371/journal.pone.0320450 (PMC11940735; doi:10.1371/journal.pone.0320450)
Supplement: S3 Table — (DOCX) [file pone.0320450.s004.docx]

**Table 3**: Transformations for the smoking variable

| Source categories | New categories |
| --- | --- |
| Does not smoke | Does not smoke |
| Less than 1 a day | Less than 1 a day |
| 1 to 5 per day | 1 to 10 per day |
| 6 to 10 per day |  |
| 11 to 20 per day | 10+ per day |
| 21 to 30 per day |  |
| 31 or more |  |
| Pipe, Cigars only | Others/Unknown |
