# Supplementary material for: Sociodemographic and early-life predictors of being overweight or obese in a middle-aged UK population– A retrospective cohort study of the 1958 National Child Development Survey participants
Source: PLoS One. 2025 Mar 26;20(3):e0320450. doi: 10.1371/journal.pone.0320450 (PMC11940735; doi:10.1371/journal.pone.0320450)
Supplement: S2 Text — (DOCX) [file pone.0320450.s005.docx]

Method of Actual Delivery: The method of actual delivery of the CM was originally coded across ~~m~~any categories, and this has been re-grouped preserving only the ones of interest, i.e., Caesarean-Elect, Caesarean-Labour, Vertex & Hand as individual groups and grouping all the rest into ‘Others’.
