# Supplementary material for: Sociodemographic and early-life predictors of being overweight or obese in a middle-aged UK population– A retrospective cohort study of the 1958 National Child Development Survey participants
Source: PLoS One. 2025 Mar 26;20(3):e0320450. doi: 10.1371/journal.pone.0320450 (PMC11940735; doi:10.1371/journal.pone.0320450)
Supplement: S3 Text — (DOCX) [file pone.0320450.s006.docx]

Father, male head’s socio-economic group (GRO 1966): The socio-economic group of the cohort member’s father or male family head, per the GRO 1966, was re-categorised.
