# Supplementary material for: Sociodemographic and early-life predictors of being overweight or obese in a middle-aged UK population– A retrospective cohort study of the 1958 National Child Development Survey participants
Source: PLoS One. 2025 Mar 26;20(3):e0320450. doi: 10.1371/journal.pone.0320450 (PMC11940735; doi:10.1371/journal.pone.0320450)
Supplement: S4 Table — (DOCX) [file pone.0320450.s007.docx]

**Table 4**: Transformation of fathers occupation

| Source category | New Category |
| --- | --- |
| Agric worker | Farm |
| Skilled manual | Manual |
| Emp,manag,small | Professional |
| Intermed non-man |  |
| Semi skld manual | Manual |
| Emp,manag,large |  |
| Foremen-manual |  |
| Junior non-man | Professional |
| Armed forces | Professional |
| Work-own account | Professional |
| Prof-employees | Professional |
| Farm-own account | Farm |
| Unskilled manual | Manual |
| Prof-self-emp | Professional |
| Farmer-emp,manag | Farm |
| Personal service | Professional |
