# Supplementary material for: Sociodemographic and early-life predictors of being overweight or obese in a middle-aged UK population– A retrospective cohort study of the 1958 National Child Development Survey participants
Source: PLoS One. 2025 Mar 26;20(3):e0320450. doi: 10.1371/journal.pone.0320450 (PMC11940735; doi:10.1371/journal.pone.0320450)
Supplement: S4 Text — (DOCX) [file pone.0320450.s008.docx]

Mother’s BMI at birth

The weight and height of the CM’s mother at CM’s birth were used to calculate the BMI of the mother. However, the weight in stones was encoded in such a way that each value represents an interval of weights rather than a point measurement. The mid-point of this interval was used for the BMI calculation.
