# Supplementary material for: Sociodemographic and early-life predictors of being overweight or obese in a middle-aged UK population– A retrospective cohort study of the 1958 National Child Development Survey participants
Source: PLoS One. 2025 Mar 26;20(3):e0320450. doi: 10.1371/journal.pone.0320450 (PMC11940735; doi:10.1371/journal.pone.0320450)
Supplement: S5 Text — (DOCX) [file pone.0320450.s009.docx]

Current Job – Social Class

The dataset for sweep 6 contains the job category of the CM at age 42, for which the source categories have been renamed/regrouped.
