# Supplementary material for: Sociodemographic and early-life predictors of being overweight or obese in a middle-aged UK population– A retrospective cohort study of the 1958 National Child Development Survey participants
Source: PLoS One. 2025 Mar 26;20(3):e0320450. doi: 10.1371/journal.pone.0320450 (PMC11940735; doi:10.1371/journal.pone.0320450)
Supplement: S5 Table — (DOCX) [file pone.0320450.s010.docx]

**Table 5**: Transformations for job categories

| Source category | New Category |
| --- | --- |
| I Professional | Professional/Technical/Non-Manual |
| II Managerial-technical |  |
| IIINM Skilled non-manual |  |
| IIIM Skilled manual | Skilled/Unskilled Manual |
| IV Partly skilled |  |
| V Unskilled |  |
| Others | Others |
