# Supplementary figures and images for: Sociodemographic and early-life predictors of being overweight or obese in a middle-aged UK population– A retrospective cohort study of the 1958 National Child Development Survey participants
Source: PLoS One. 2025 Mar 26;20(3):e0320450. doi: 10.1371/journal.pone.0320450 (PMC11940735; doi:10.1371/journal.pone.0320450)

**Figure 1**: Comparison of BMI categories


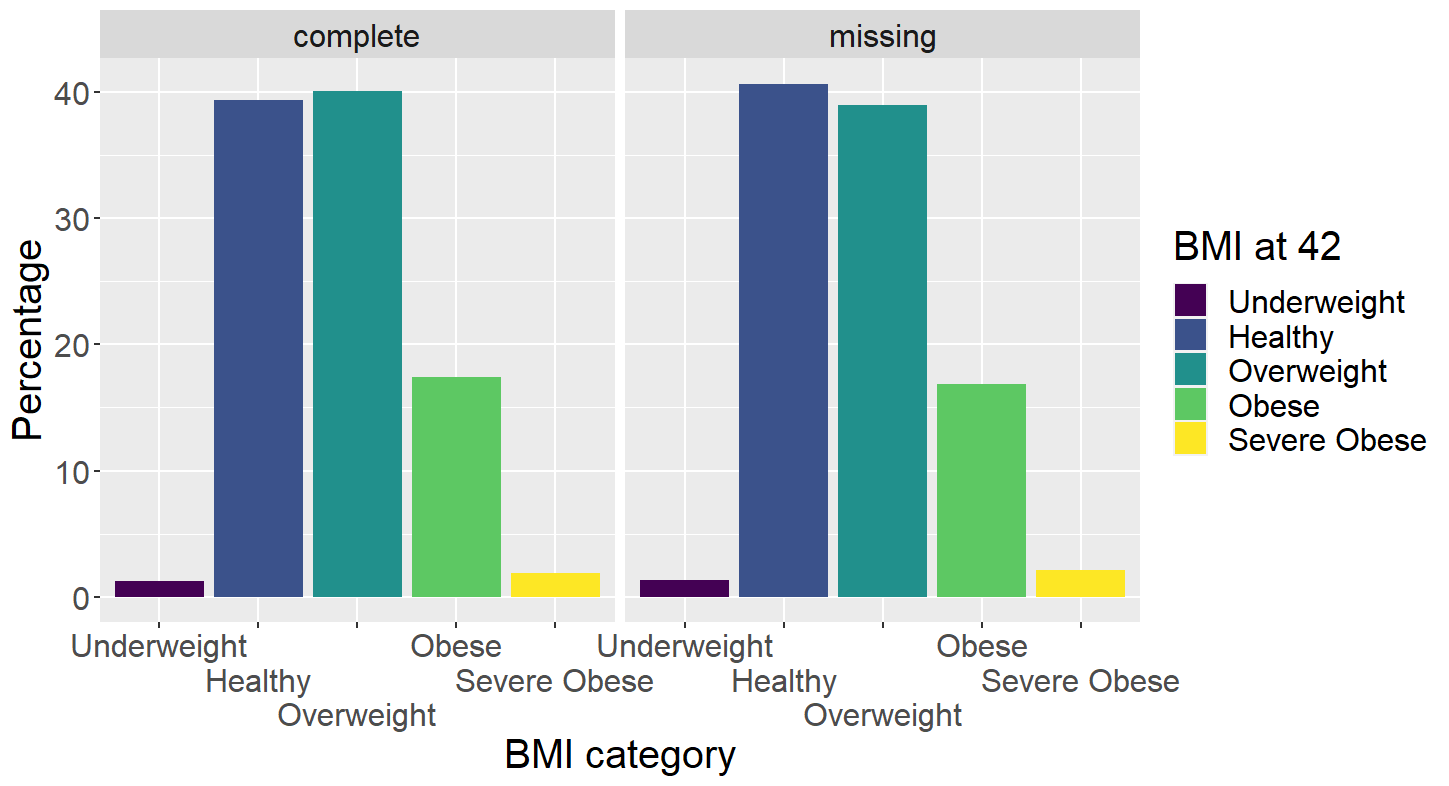

Supplement: S1 Fig — (DOCX) [file pone.0320450.s011.docx]

**Figure 2**: Comparison of exercise frequency

 
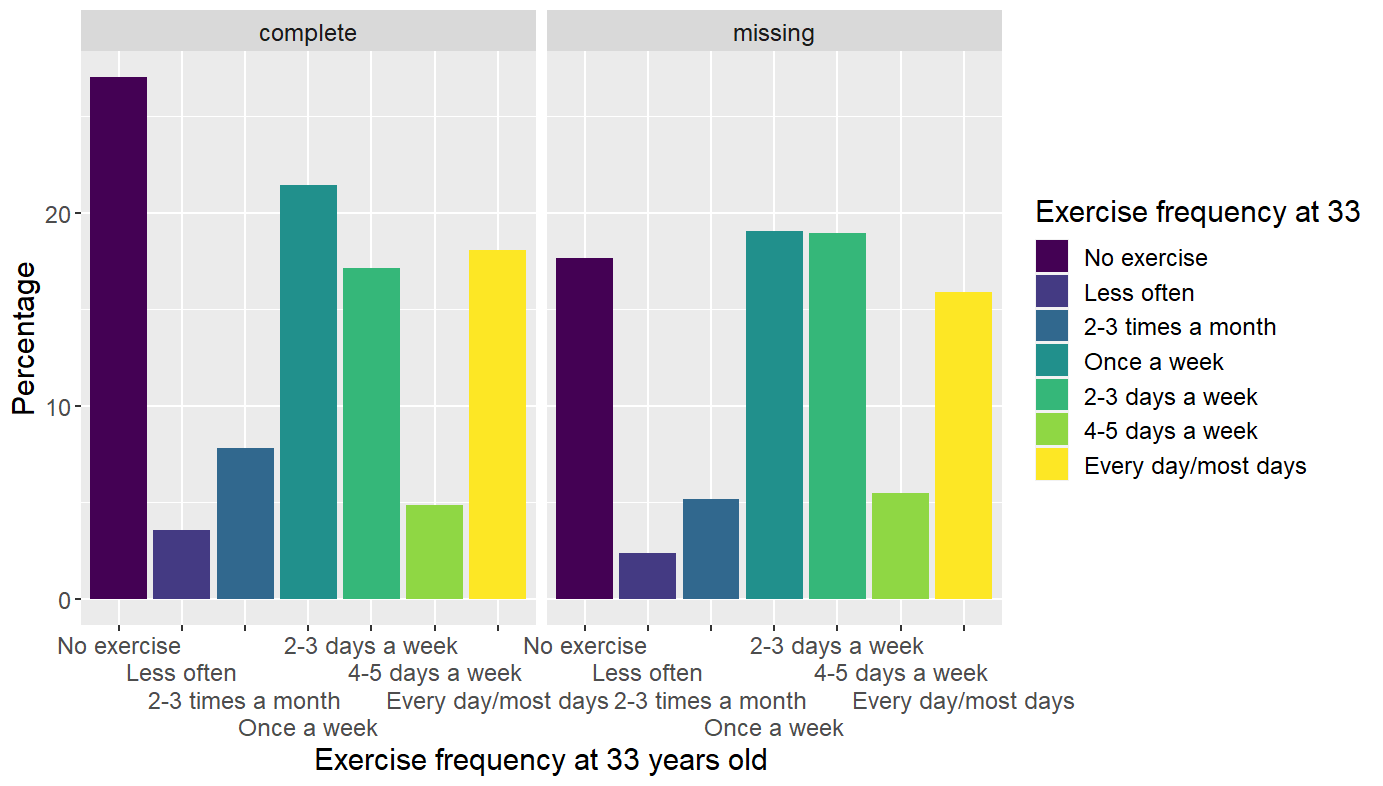

Supplement: S2 Fig — (DOCX) [file pone.0320450.s012.docx]

**Figure 3**: Comparison of mother’s BMI


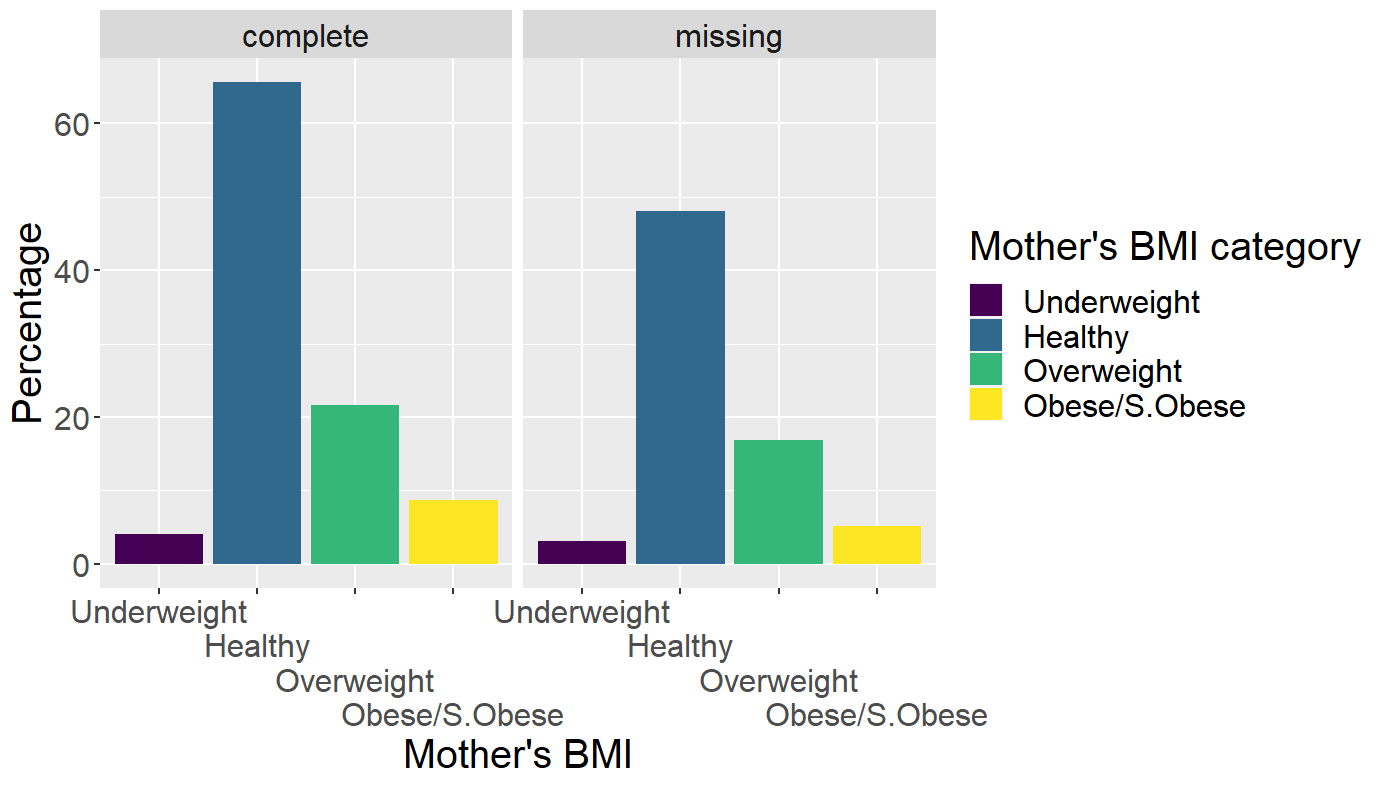

Supplement: S3 Fig — (DOCX) [file pone.0320450.s013.docx]
