# Supplementary material for: Sociodemographic and early-life predictors of being overweight or obese in a middle-aged UK population– A retrospective cohort study of the 1958 National Child Development Survey participants
Source: PLoS One. 2025 Mar 26;20(3):e0320450. doi: 10.1371/journal.pone.0320450 (PMC11940735; doi:10.1371/journal.pone.0320450)
Supplement: S6 Table — (DOCX) [file pone.0320450.s014.docx]

**Table 6**: Model Diagnostics

| Model | AUC-ROC | Nagelkerke R² | AIC | BIC |
| --- | --- | --- | --- | --- |
| M1 | 0.64 | 0.066 | 6,560 | 6,711 |
| M2 | 0.63 | 0.054 | 3,964 | 4,106 |
| M3 | 0.71 | 0.090 | 3,158 | 3,271 |
