# Supplementary material for: Sociodemographic and early-life predictors of being overweight or obese in a middle-aged UK population– A retrospective cohort study of the 1958 National Child Development Survey participants
Source: PLoS One. 2025 Mar 26;20(3):e0320450. doi: 10.1371/journal.pone.0320450 (PMC11940735; doi:10.1371/journal.pone.0320450)
Supplement: S7 Table — (DOCX) [file pone.0320450.s015.docx]

**Table 7**: Results for the full model for O42 (with all variables)

| **Characteristic** | **OR**^1^ | **95% CI**^1^ | **p-value** |
| --- | --- | --- | --- |
| **Exercise Frequency at 33** |  |  |  |
| No exercise | — | — |  |
| Less often | 0.887 | 0.579, 1.327 | 0.571 |
| 2-3 times a month | 0.815 | 0.607, 1.085 | 0.167 |
| Once a week | 0.825 | 0.678, 1.004 | 0.055 |
| 2-3 days a week | 0.646 | 0.527, 0.791 | **<0.001** |
| 4-5 days a week | 0.533 | 0.382, 0.733 | **<0.001** |
| Every day/most days | 0.752 | 0.610, 0.925 | **0.007** |
| **Job category at 42** |  |  |  |
| Others | — | — |  |
| Professional/Technical/Non-Manual | 0.671 | 0.550, 0.821 | **<0.001** |
| Skilled/Unskilled Manual | 0.866 | 0.701, 1.073 | 0.187 |
| **smoking_42** |  |  |  |
| Never Smoked Cigarettes, | — | — |  |
| Used To Smoke But Dont At All Now | 1.149 | 0.977, 1.350 | 0.091 |
| Smoke Cigarettes Occasionally | 0.928 | 0.649, 1.302 | 0.675 |
| Smoke Cigarettes Every Day | 0.726 | 0.606, 0.868 | **<0.001** |
| **Mother's smoking at birth** |  |  |  |
| Does not smoke | — | — |  |
| Less than 1 a day | 0.735 | 0.444, 1.164 | 0.208 |
| 1-10 per day | 1.363 | 1.095, 1.692 | **0.005** |
| 10+ per day | 1.480 | 1.227, 1.783 | **<0.001** |
| Unknown/Others | 1.153 | 0.904, 1.473 | 0.253 |
| **Father's smoking at birth** |  |  |  |
| Does not smoke | — | — |  |
| Less than 1 a day | 1.089 | 0.554, 1.994 | 0.793 |
| 1-10 per day | 1.189 | 0.924, 1.521 | 0.174 |
| 10+ per day | 1.316 | 1.092, 1.587 | **0.004** |
| Unknown/Others | 1.112 | 0.879, 1.402 | 0.374 |
| **Delivery Method** |  |  |  |
| Vertex and hand | — | — |  |
| Caesarean-labour | 1.871 | 1.112, 3.059 | **0.015** |
| Caesarean-elect | 1.256 | 0.663, 2.247 | 0.462 |
| Others | 1.169 | 0.944, 1.439 | 0.147 |
| **Sex** |  |  |  |
| Male | — | — |  |
| Male | — | — |  |
| Female | 0.942 | 0.818, 1.084 | 0.402 |
| **Mother's BMI at CM's birth** |  |  |  |
| Healthy | — | — |  |
| Obese/Severe Obese | 2.930 | 2.333, 3.671 | **<0.001** |
| Overweight | 1.945 | 1.667, 2.267 | **<0.001** |
| Underweight | 0.811 | 0.542, 1.179 | 0.291 |
| **Father's BMI** |  |  |  |
| Healthy | — | — |  |
| Obese/Severe Obese | 2.476 | 1.896, 3.218 | **<0.001** |
| Overweight | 1.526 | 1.325, 1.758 | **<0.001** |
| Underweight | 1.915 | 1.033, 3.369 | **0.030** |
| **1P Child's position in birth order** | 0.971 | 0.919, 1.024 | 0.282 |
| **Mother's age at CM's birth** | 0.989 | 0.975, 1.004 | 0.144 |
| **Gestation period in days** | 1.000 | 0.994, 1.006 | 0.961 |
| **Breastfed** |  |  |  |
| Dont know | — | — |  |
| Dont know | — | — |  |
| No | 2.413 | 0.617, 16.06 | 0.265 |
| Under one month | 2.177 | 0.555, 14.51 | 0.326 |
| Over one month | 2.080 | 0.532, 13.84 | 0.354 |
| **Father's Social Group (1966)** |  |  |  |
| Farm | — | — |  |
| Manual | 0.958 | 0.790, 1.160 | 0.663 |
| Others | 0.979 | 0.717, 1.321 | 0.893 |
| Professional | 0.824 | 0.704, 0.963 | **0.015** |
| **Birthweight in ounces** | 1.005 | 1.001, 1.010 | **0.025** |
| **Birthweight - Gestational Age for Sex** |  |  |  |
| Standard | — | — |  |
| Low (<-2SD) | 1.154 | 0.619, 2.041 | 0.636 |
| High (>+2SD) | 1.159 | 0.766, 1.731 | 0.477 |
| Others | 0.801 | 0.380, 1.553 | 0.534 |
| ^1^OR = Odds Ratio, CI = Confidence Interval | | | |
